# The Teaching of Elementary Science

**Published:** 1898-05-28

**Authors:** 


### THE TEACHING OF ELEMENTARY SCIENCE.

The resolution of the Royal College of Physicians in regard to the teaching of elementary scientific subjects is likely to land the medical schools in a very considerable difficulty. According to the proposed arrangement the first of the five years required to be devoted to professional study may be spent in obtaining instruction in chemistry, physics, practical chemistry, and biology, either at a recognised institution (*i.e.*, a place other than a medical school) or at a recognised medical school, leaving only four years which must elapse between joining the medical school and entering for the final examination; and it is only to be expected that a very large proportion of the students of the future will elect to get rid of these subjects before going up to the hospitals. Under these circumstances, the medical schools will have to consider whether it will be worth their while to keep up their laboratories and all the expensive machinery which is required for the teaching of these subjects to the small residuum of students who will enter for the first year. Much will depend upon the number of "institutions" which are "recognised" as places of study. But if technical schools and higher-grade Board Schools are to be recognised, as has been suggested, it is clear that the economy of getting through their chemistry, physics, practical chemistry, and biology before joining will be so great that but few will be left to repay the medical schools for their outlay on the teaching of these subjects. A great opportunity, then, is likely to offer itself to the medical schools of London to get rid of a quantity of useless top-hamper in the way of physics, biology, and chemistry teaching, if they will but combine so to do. Unfortunately, the trustful parent has to be impressed by the prospectus, and it is to be feared that schools will hesitate to admit that they are not complete. With the steady growth and importance, however, of bacteriology and of physiological and pathological chemistry, the space at the disposal of the schools might well be devoted to these higher branches, and some form of combination school might be formed, at which the elementary science subjects might be taught to such students as had not been connected with "recognised institutions." Something of this sort would clear the board immensely, and would be one step towards leaving the medical schools none but medical matters to deal with.

